# Sympatric competitors have driven the evolution of temporal activity patterns in *Cnemaspis* geckos in Southeast Asia

**DOI:** 10.1038/s41598-019-56549-x

**Published:** 2020-01-08

**Authors:** Hung Ngoc Nguyen, Chih-Ming Hung, Ming-Yuan Yang, Si-Min Lin

**Affiliations:** 1 Department of Zoology, Southern Institute of Ecology, Vietnam Academy of Science and Technology, Ho Chi Minh City, Vietnam; 2 School of Life Science, National Taiwan Normal University, Taipei, Taiwan; 3 Biodiversity Program, Taiwan International Graduate Program, Academia Sinica, Taipei, Taiwan; 4 Biodiversity Research Center, Academia Sinica, Taipei, Taiwan

**Keywords:** Evolutionary ecology, Evolutionary theory, Phylogenetics

## Abstract

It is often assumed that animals’ temporal activity patterns are highly conserved throughout evolution. While most geckos are nocturnal, the species in the *Cnemaspis* genus are mostly diurnal (only a few are nocturnal). This raises a question about the evolution of a diel niche in the *Cnemaspis* genus. *Cnemaspis* geckos are distributed across Southeast Asia and are often sympatric with *Cyrtodactylus*, another widespread gecko genus in the same area. Since both genera are mainly rocky habitat specialists, we hypothesize that *Cyrtodactylus* may influence the temporal activity pattern of *Cnemaspis* when they are sympatric through competition. By analyzing habitat data, diel activity, and the existence of sympatric *Cyrtodactylus* species across the phylogeny of the *Cnemaspis* genus, we found (1) strong phylogenetic signals in the habitat use trait but not in temporal activity, suggesting that the diel niche of this genus is more labile compared with habitat niche, and (2) a significant association with the temporal activity pattern of *Cnemaspis* and the sympatry between the two genera, with the former tending to be diurnal when they are sympatric. Originated from a diurnal common ancestor, the release from competition with *Cyrtodactylus* species might open an opportunity for some *Cnemaspis* species to shift to nocturnal niches.

## Introduction

Niche partition among sympatric species is an important mechanism to determine species richness in a community^1–3^. Although there have been great efforts to study how sympatric species separate their ecological niches along the spatial dimension (e.g.^4,5^), we still know little about their diel niche segregation^6^. Many sympatric taxa that share habitats or food resources might be active at different time periods to avoid competition^7^. However, studies focusing on the diel niche transitions are extremely rare. A good system for such research requires a suitable taxonomic group with a variety of temporal activity patterns, well-resolved phylogenetic relationships, and reliable ecological data.

Based on phylogenetic analyses of diel niche evolution, Anderson and Wiens^8^ suggested that terrestrial vertebrates may have nocturnal ancestors and show strong phylogenetic signals in this trait. They further supported the idea presented by Vitt *et al*.^9^ that the temporal niche partition among current species has been largely conserved over evolution. Among current nocturnal vertebrates, geckos belong to a taxonomic group containing primarily nocturnal species with a nocturnal ancestor^10^. However, Gamble and colleagues^10^ found that there were multiple transitions between diurnality and nocturnality in this group during various time periods including several recent ones. Contradicting with the strong conservatism in temporal activity pattern in most vertebrates, the recent shifts between nocturnality and diurnality in geckos provide good chances for testing temporal niche partitioning and ecological community assembly.

Southeast Asia *Cnemaspis* (rock geckos), composed of 55 species^11,12^, is a speciose genus in gekkonid lizards. This genus forms a large proportion of species that are endemic to extremely limited distribution ranges^12^. As rocky habitat specialists, these species tend to maintain strong niche conservatism through a long divergence history^13^; the high species richness of this genus is thus regarded as a consequence of their strict specialization in fragmented rocky habitats. However, this genus is composed of a majority of diurnal species and a minority of nocturnal species^12^, implying that they might have experienced multiple diel transitions.

Interestingly, *Cnemaspis* species are usually sympatric with another nocturnal gecko genus *Cyrtodactylus*^12^, which comprises more than 200 species and is the most speciose gekkonid group across Southeast Asia^14–16^. Similar to *Cnemaspis*, many *Cyrtodactylus* geckos are strongly specialized in rocky habitats^17–19^. The typical habitats of these two genera of geckos, such as rocks, karst topology, or boulder caves, are usually extremely limited in space and food resources. Under this situation, *Cnemaspis* geckos might face strong competition from *Cyrtodactylus* geckos when they are sympatric. Since there are many records that diurnal *Cnemaspis* geckos are co-distributed with nocturnal *Cyrtodactylus* species^12,20,21^, we hypothesize that a diel niche partitioning between these two geckos may have evolved to avoid competition.

In this study, we performed phylogenetic comparative analyses on habitat use and temporal activity patterns of the *Cnemaspis* genus to identify their ancestral states of these traits along the phylogeny. We aim to (1) infer if there is niche conservatism or transition in these traits; (2) examine the correlation between the temporal activity patterns and sympatry of *Cnemaspis* and *Cyrtodactylus* species; and (3) determine other potential ecological or environmental factors that impact the temporal activity patterns of *Cnemaspis* geckos. This will be one of the first studies to examine the interaction between biological competition and diel niche evolution in reptiles.

## Results

**Figure 1:**
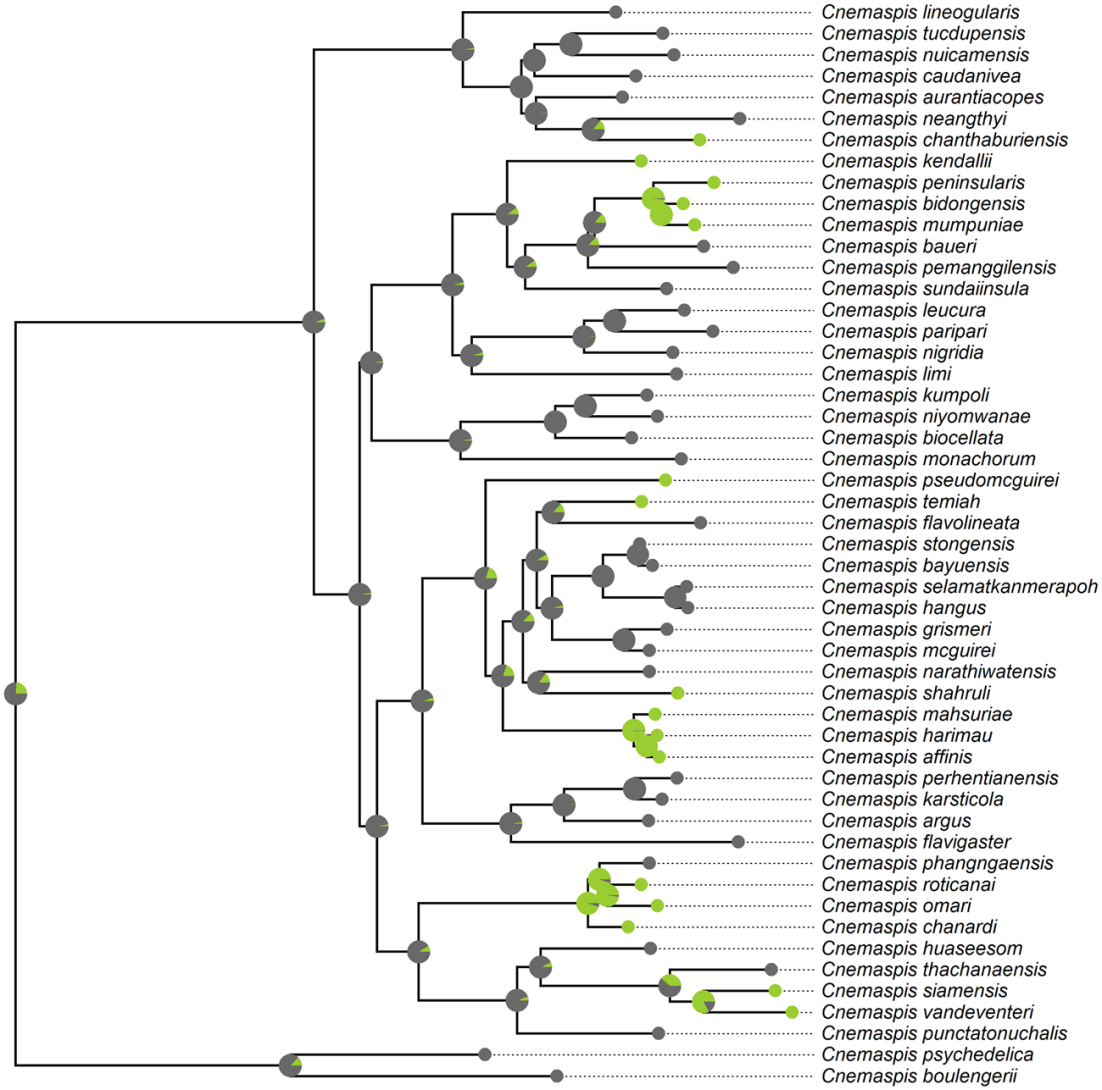
The evolution of habitat use in *Cnemaspis* genus. Bayesian ancestral state reconstructions of habitat use were mapped onto the mitochondrial ND2 phylogeny of *Cnemaspis* using the asymmetric multi-rate model. Circles at the tips of branches indicate the habitat use type for each included species. Pie charts on internal nodes indicate the posterior probability of that ancestor having a particular habitat use type. Species are categorized as rocky habitat specialists (grey) and habitat generalist/other habitat specialists (green).

**Figure 2:**
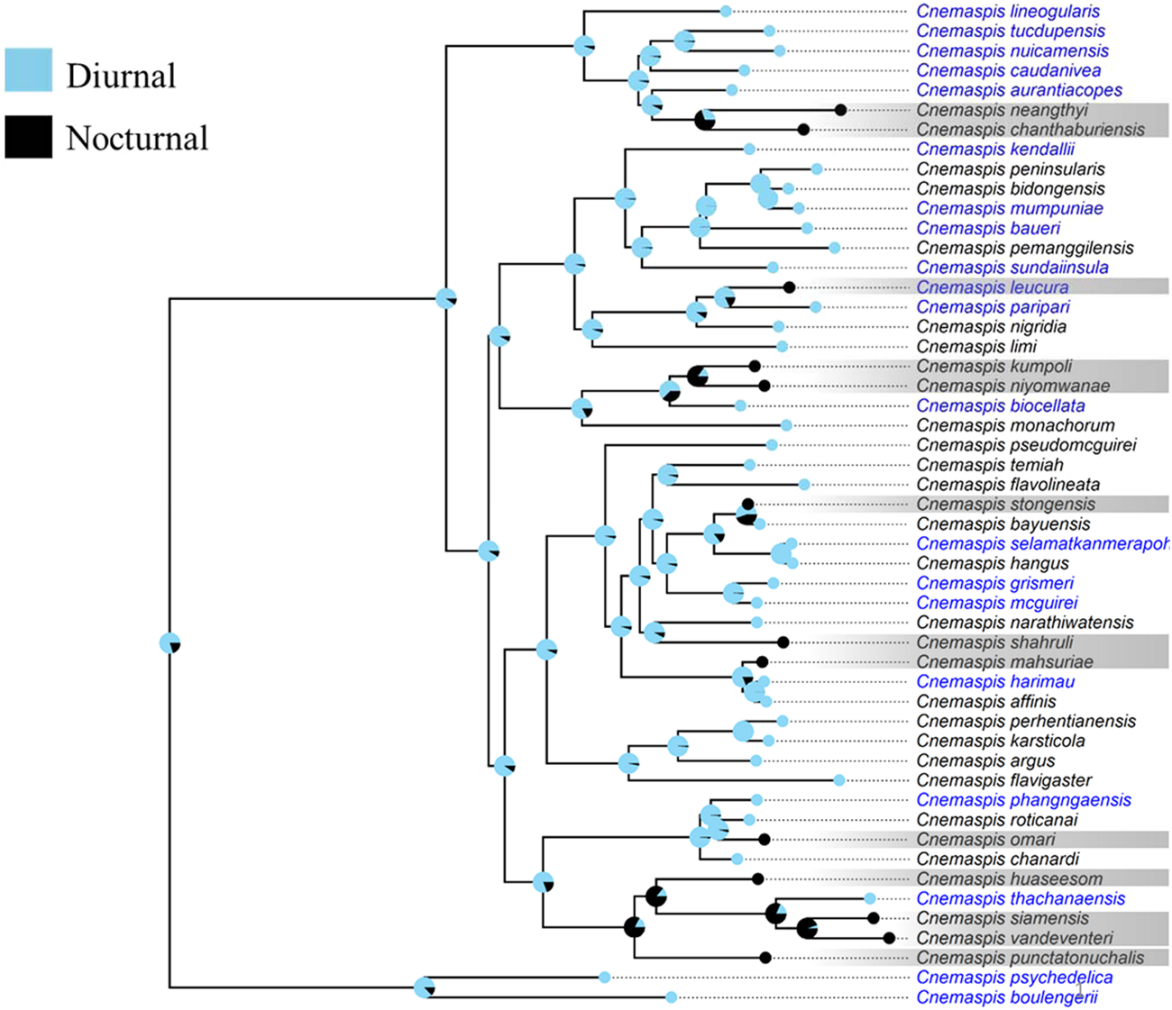
The evolution of temporal niche in *Cnemaspis* genus. Bayesian ancestral state reconstructions of temporal activity were mapped onto the mitochondrial ND2 phylogeny of *Cnemaspis* using the asymmetric multi-rate model. Circles at the tips of branches indicate the temporal niche for each included species. Pie charts on internal nodes indicate the posterior probability of that ancestor having a particular temporal niche. Species are categorized as nocturnal (black) and diurnal (blue). The species names in blue color indicate the existence of sympatric *Cyrtodactylus* species.

There are 51 *Cnemaspis* species with available ND2 sequences from GenBank, representing 93% among the 55 currently recognized species (Table S1). Among them, 36 were diurnal and 15 were nocturnal. About two-thirds of them were specialists in rocky habitats (35 species) while others (16 species) were terrestrial or habitat generalists. Among them, 20 species were sympatric with *Cyrtodactylus* species (Table S2). The original phylogeny of *Cnemaspis* with outgroup species is presented in Fig. S1. Phylogenies with ecological character states are demonstrated in Figs. 1 and 2.

### Phylogenetic conservation

The habitat and diel activity niches of the *Cnemaspis* species showed different levels of phylogenetic signals (Table 1). The estimated D ≈ 0.299 of habitat use suggested a high level of phylogenetic signal in this trait. The simulation test indicated that the level of phylogenetic signal differed significantly from that of a random phylogenetic structure but was consistent with the expectation of Brownian motion. In contrast, the estimated D ≈ 0.789 of the temporal activity pattern indicated a low level of phylogenetic signal. The simulation test on temporal activity showed that the phylogenetic signal level was consistent with the expectation of a random phylogenetic structure.

**Table 1:** Estimated phylogenetic signal strength (D) for the habitat type and temporal activity mode for *Cnemaspis* genus.

	Habitat type	Temporal activity
Estimated D	0.2989471	0.7895734
Random Phylogenetic Structure (p value)	<0.01	0.228
Brownian Phylogenetic Structure (p value)	0.221	0.018

### Ancestral state reconstruction

For both temporal activity and habitat use traits, the transition rates between character states based on single- and two-rate models in BayesTraits did not show a significant difference (temporal activity: single rate = −33.82 and asymmetric rates model = −31.33, logBF = 4.977; habitat use: single rate = −29.25 and asymmetric rates model = −28.29, logBF = 1.933). Both transition rate models favored a diurnal and rocky-specialist common ancestor for the *Cnemaspis* genus (Table 2).

**Table 2:** Estimated probability of ancestral state at the most recent common ancestor of *Cnemaspis* genus using BayesTraits v2.0.

Model	Ancestral state	Mean	95% HPD Interval
**(a) Temporal activity: D — diurnal**, **N — nocturnal**			
Asymmetric rate	Root P(D)	0.503	[0.4984, 0.5139]
	Root P(N)	0.497	[0.4862, 0.5016]
Equal rate	Root P(D)	0.625	[0.5000, 0.8631]
	Root P(N)	0.375	[0.1369, 0.5000]
**(b) Habitat use: O — other habitat/generalist**, **R — rock habitat**			
Asymmetric rate	Root P(O)	0.4184	[0.2324, 0.5000]
	Root P(R)	0.5816	[0.5000, 0.7676]
Equal rate	Root P(O)	0.2088	[7.4 × 10^−5^, 0.4571]
	Root P(R)	0.7912	[0.5429, 0.9999]

The stochastic mapping analyses showed that the asymmetric multi-rate model was preferred (Table S3). The results of ancestral state reconstruction based on the stochastic mapping approach were congruent with those of the Bayestraits analyses (Figs. 1 and 2), suggesting the robustness of these results.

### Regression between temporal activity modes and ecological factors

The best model included the existence of sympatric *Cyrtodactylus* species, along with the mean diurnal range and precipitation of the driest quarter (Tables 3 and 4), together explained 48.1% of the temporal activity patterns in the *Cnemaspis* genus. Although the mean diurnal range parameter (bio2) was included in the best model, this parameter was weakest, not significant factor, and the performance of the model was slightly decreased without it (Table 3).

**Table 3:** Akaike information criterion (AIC) for model selection.

Model	Df	AIC
Active_time ~ Cyrto_Sym + bio2 + bio17*	47	45.75073
Active_time ~ Cyrto_Sym + bio17	48	46.69000
Active_time ~ Cyrto_Sym + dist_range + bio2 + bio17	46	46.86641
Active_time ~ Cyrto_Sym + dist_range + bio2 + bio17 + bio18	45	48.3432
Active_time ~ Cyrto_Sym + max_SVL + dist_range + bio2 + bio17 + bio18	44	49.41486
Active_time ~ bio2 + bio17	48	50.30900
Active_time ~ Cyrto_Sym + max_SVL + dist_range + Avg_night_temp + bio2 + bio17 + bio18	43	51.08088
Active_time ~ Cyrto_Sym + bio2	48	51.91400
Active_time ~ Cyrto_Sym + max_SVL + dist_range + Avg_night_temp + bio2 + bio4 + bio17 + bio18	42	53.05279
Active_time ~ Cyrto_Sym	49	53.32100
Active_time ~ Cyrto_Sym + max_SVL + Habitat.type + dist_range + Avg_night_temp + bio2 + bio4 + bio17 + bio18	41	55.04756
Active_time ~ bio2	49	56.18600
Active_time ~ bio17	49	56.45700
Active_time ~ Cyrto_Sym + Habitat.type + max_SVL + dist_range + Avg_night_temp + bio2 + bio4 + bio12 + bio17 + bio18	40	57.04461

^*^The best model.

**Table 4:** Parameter estimates for the regression models of temporal activity on the ecological factors.

	Slope	Std. Error	z value	Pr(>|z|)
Sympatric *Cyrtodactylus* present	2.53E + 00	1.19E + 00	2.127	0.0334*
Mean diurnal range	8.93E − 01	5.64E − 01	1.584	0.1132
Precipitation of Driest Quarter	−8.34E − 04	3.33E − 03	−2.506	0.0122*

R^2^ model = 0.481. *p < 0.05.

## Discussion

### Temporal niche responses to competition and environmental factors

With the existence of sympatric *Cyrtodactylus* species, the mean diurnal range and precipitation of the driest quarter are the factors in the best-supported model that explain the temporal activity patterns of *Cnemaspis* genus. Our study might present the first evidence supporting that the evolution of a diel niche of a vertebrate species can be influenced by a sympatric, ecologically similar species. The absence of *Cyrtodactylus* geckos in the distribution range increases the chance that *Cnemaspis* species are active at night. Except for *Cnemaspis leucura*, almost all nocturnal *Cnemaspis* species are not sympatric with *Cyrtodactylus* species. Considering that the most recent common ancestor of *Cnemaspis* species was diurnal, the recent shift to nocturnality of these species may be the result of ecological release^22^ from the competition of *Cyrtodactylus* species. Nonetheless, we also notice that some *Cnemaspis* species without sympatric *Cyrtodactylus* are diurnal (Fig. 2). This pattern might be caused by incomplete records. Firstly, even though several new species of the *Cyrtodactylus* genus have been described in south Thailand and Malaysia^23–26^, there is still insufficient information about the fine-scaled distribution of some *Cyrtodactylus* species, especially for those potentially new ones. Secondly, competition from other geckos, including other sympatric *Cnemaspis* congeners, might also led to the temporal niche partition, leading to the somewhat inconsistent relationship between diel niche and sympatry between *Cnemaspis* and *Cyrtodactylus* species. For example, it has been reported that *Cnemaspis* congeners could have sympatric distribution and temporal niche partition (*C*. *kendallii* versus *C*. *nigridia*, *C*. *leucura* versus *C*. *kendallii* and *C*. *monachorum* versus *C*. *roticanai*^12,27,28^). Furthermore, competition pressure might also come from nocturnal geckos other than *Cyrtodactylus* species, as they are abundant in Southeast Asia^29^.

Our results are consistent with those of Vidan *et al*.^29^ in supporting the precipitation of the driest quarter as a strong predictor for the distribution of nocturnal lizards. The negative relationship between the rainfall in dry quarters and the nocturnal activity levels of *Cnemaspis* species indicates that the geckos tend to be more active at night when the dry season is drier. This might be related to the water retention of the geckos; they have higher rates of water loss than other lizards^30^ and the water loss rate increases with ambient temperature^31^. Therefore, *Cnemaspis* species tend to be nocturnal in arid habitats to avoid high water loss. Moreover, in accordance with the results of Cunningham *et al*.^32^ and Vidan *et al*.^29^, the mean diurnal range is also determined to be one factor in our best model. However, this parameter does not seem to contribute much to the model since the support of our model is slightly decreased without it. Furthermore, despite the positive relationship with nocturnal activity levels, the *Cnemaspis* species with the highest diurnal range are still active at day time (Table S2).

### Conservatism in temporal and spatial niches

In contrast to Anderson and Wiens^8^, who suggest that diel activity patterns have long-term conservatism, our results show little phylogenetic signal or conservatism in the temporal activity pattern of *Cnemaspis* geckos. However, we find strong niche conservatism in the habitat use of *Cnemaspis* geckos. These results suggest that *Cnemaspis* might change their active times more frequently than their habitat use. Theoretically, the pupil of geckos could be categorized into vertical narrow pupils for nocturnal taxa and circular ones for diurnal taxa^33^. However, the real relationship between pupil shapes and temporal active modes is controversial^34^. Although all species of *Cnemaspis* have circular pupils, multiple species in this genus are nocturnal. The labile diel niche *Cnemaspis* geckos might reflect the history that they could have evolved from a nocturnal ancestor that predated the diurnal one (i.e., their most recent common ancestor) inferred in this study, and such shift might have occurred repeatedly over their evolution. In addition, many diurnal geckos (e.g., *Sphaerodactylus macrolepis*) can be active in dim light conditions^35^. Some diurnal lizards can even opportunely switch to a nocturnal mode to avoid predators or competition, or to utilize novel resources^36^. Therefore, diurnal *Cnemaspis* species, which are likely used to low light conditions since they mostly restrict their movements to the shadow under rocks, should be able to easily change to a night active mode. Such adaptive flexibility may explain their labile temporal active patterns.

The diurnal ancestral state of the *Cnemaspis* genus, which belongs to a lizard groups with mostly nocturnal lineages^10^, suggests that the ancestor of this genus might have used a day time niche in order to avoid competition from other sympatric nocturnal geckos^29^. Moreover, their ancestral state of habitat use was the rocky habitat that often has limited space and resources, and thus might drive the diel niche partition among sympatric geckos to avoid direct competition. Our analyses of temporal activity pattern and habitat use across the *Cnemaspis* phylogeny suggest that the *Cnemaspis* species are more likely to change their active time than their habitat use to avoid competition over evolution. Given that nocturnal *Cnemaspis* species mostly occur exclusively where there is no existence of sympatric *Cyrtodactylus* species, the former might be inferior competitors than the latter. Therefore, the empirical evidence found in this study supports that the recent shifts to nocturnality in *Cnemaspis* species might be the result of ecological release from the competition of *Cyrtodactylus* species.

## Methods

### Phylogenetic relationship

The mitochondrial ND2 sequences of 51 *Cnemaspis* species (Table S1) were retrieved from GenBank and aligned using CLUSTAL^37^ implemented in MEGA 6^38^. We estimated the phylogenetic relationships among *Cnemaspis* species in a Bayesian framework using MrBayes v3.2.6^39^. The optimal model of sequence evolution (GTR + I + G) was determined using Bayesian information criterion (BIC) in jModelTest 2.1.8^40^. We ran two independent Metropolis-coupled MCMC analyses; each one was run for 2 million generations, with sampling every 1000 generations. Thirty-one species from *Alsophylax*, *Gehyra*, *Hemiphyllodactylus*, *Microgecko*, and *Perochirus* genera were used as outgroups (Table S1).

### Ecology data collection

The life history information of each *Cnemaspis* species, including temporal activity pattern, maximum body size (SVL), habitat use, and the existence of sympatric *Cyrtodactylus* species, was retrieved from the published literature (i.e.^12,41,42^) (Table S2). For the temporal activity pattern, we defined the study species as “diurnal” or “nocturnal” based on the description of their active time in the literature. The existence of sympatric *Cyrtodactylus* species was indicated by two states (yes/no). For the main habitat type, species that specialized in rocky habitat were recorded as “rocks”, and those used all other habitats (terrestrial or habitat generalists) were recorded as “others”. For the dispersal potential, the distances between all localities of each species were calculated using the distm function of the R package “geosphere”^43^, and the maximum distance was selected as the representative dispersal potential of each species.

Following the results of Cunningham *et al*.^32^ and Vidan *et al*.^29^, we chose the following seven climate variables that strongly effect the distribution of reptiles and diurnal lizards from WorldClim version 2^44^: annual mean temperature (bio1); mean diurnal range (bio2); temperature seasonality (bio4); mean temperature of coldest quarter (bio6); precipitation seasonality (bio12); precipitation of driest quarter (bio17); and precipitation of warmest quarter (bio18). The variables were extracted from 2.5-minute resolution layers of WorldClim version 2^44^. Furthermore, we included night temperature from the Moderate Resolution Imaging Spectroradiometer (MODIS) Land Surface Temperature and Emissivity dataset (MOD11A2 Terra)^45^; the values were extracted from high-resolution (~1 km) remote-sensed land surface temperature (LST) data by using the R package “MODISTools”^46^.

### Phylogenetic conservation

We used the method developed by Fritz and Purvis^47^, which is specific for measuring phylogenetic signals in binary traits, to estimate the character dispersion on a phylogeny (D) using the R package “caper”^48^. Here, a D < 0 suggests a highly clustered trait, D ~ 0 indicates that the trait is phylogenetically conserved as expected under a Brownian threshold model, D = 1 suggests that trait values are random at the tips of the phylogeny, and D > 1 suggests phylogenetic over-dispersion^47^. In order to assess significance for each trait of temporal activity and habitat use, we performed 1000 simulated permutations based on random or Brownian motion patterns of evolution and compared the observed patterns to these two distributions.

### Ancestral state reconstruction

The ancestral states of temporal activity patterns and habitat use were reconstructed using two methods: Bayesian ancestral state reconstruction and stochastic mapping. Bayesian ancestral state reconstruction was performed using BayesTraits v2.0^49^. A set of 2000 trees, drawn from the posterior distribution of trees inferred by MrBayes analyses, was used to incorporate phylogenetic uncertainty. Analyses were conducted for 1 million generations, sampled every 1000 generations, and the first 10,000 generations were discarded as burn-in. We conducted three chains of each analysis to assess convergence of the results by checking their MCMC trend lines. Models with different transition rates were built and compared based on Bayes factors.

For stochastic mapping, we mapped the temporal activity and habitat use states onto the maximum credibility tree from MrBayes analysis using the simmap function in the R package “phytools”^50^. The transition models that best fitted the data were estimated based on maximum likelihood using the ace function in the R package “APE”^51^.

### Regression between temporal active patterns and ecological factors

We used logistic regression analysis to test the relationship between temporal activity modes and the ecological factors (existence of sympatric *Cyrtodactylus* species, habitat types, dispersal potential, and climate variables). The variables were tested for collinearity examining the variance inflation factor with the vif function in the R package “car”^52^. The mean annual temperature (bio1) and mean temperature of coldest quarter (bio6) were excluded due to a high correlation between them and other climate variables. We used a backwards stepwise model selection process, and selected the best model according to Akaike Information Criterion (AIC) scores by using the stepAIC function in the R package “MASS”^53^.

## Supplementary information

Supplementary Information

## Data Availability

Data relevant to the study, including GenBank Accession numbers and tree files, are provided in Supporting Information.
