# Supplementary material for: Sympatric competitors have driven the evolution of temporal activity patterns in Cnemaspis geckos in Southeast Asia
Source: Sci Rep. 2020 Jan 8;10:27. doi: 10.1038/s41598-019-56549-x (PMC6949239; doi:10.1038/s41598-019-56549-x)
Supplement: Supplementary file 1 — Supplementary Information [file 41598_2019_56549_MOESM1_ESM.docx]

**Supplementary Information**

**Sympatric competitors have driven the evolution of temporal activity patterns in *Cnemaspis* geckos in Southeast Asia**

Hung Ngoc NGUYEN^1,2,3^, Chih-Ming HUNG^3,4^, Ming-Yuan YANG^2,3^, Si-Min LIN^2,3*^

^1^ Department of Zoology, Southern Institute of Ecology, Vietnam Academy of Science and Technology, Vietnam

^2^ School of Life Science, National Taiwan Normal University, Taipei, Taiwan

^3^ Biodiversity Program, Taiwan International Graduate Program, Academia Sinica, Taipei, Taiwan

^4^ Biodiversity Research Center, Academia Sinica, Taipei, Taiwan

*Corresponding Author: Si-Min LIN: [lizard.dna@gmail.com](mailto:lizard.dna@gmail.com)

**Running Head:** Competitors drive temporal activity patterns

**Table S1.** Species and mitochondrial sequences used in phylogenetic construction.

| Genus | Species | Genbank ID |
| --- | --- | --- |
| *Cnemaspis* | *C. affinis* | KM024685 |
|  | *C. argus* | KM024689 |
|  | *C. aurantiacopes* | KM024693 |
|  | *C. baueri* | KM024699 |
|  | *C. bayuensis* | KM024700 |
|  | *C. bidongensis* | KM024706 |
|  | *C. biocellata* | KM024709 |
|  | *C. boulengerii* | KM024710 |
|  | *C. caudanivea* | KM024712 |
|  | *C. chanardi* | KM024715 |
|  | *C. chanthaburiensis* | KM024716 |
|  | *C. flavigaster* | KM024720 |
|  | *C. flavolineata* | KM024721 |
|  | *C. grismeri* | KM024722 |
|  | *C. hangus* | KM024729 |
|  | *C. harimau* | KM024730 |
|  | *C. huaseesom* | KM024735 |
|  | *C. karsticola* | KM024737 |
|  | *C. kendallii* | KM024743 |
|  | *C. kumpoli* | KM024745 |
|  | *C. leucura* | LC158334 |
|  | *C. limi* | KM024749 |
|  | *C. lineogularis* | KY091233 |
|  | *C. mahsuriae* | KT250634 |
|  | *C. mcguirei* | KM024753 |
|  | *C. monachorum* | KM024758 |
|  | *C. mumpuniae* | KM024761 |
|  | *C. narathiwatensis* | KM024765 |
|  | *C. neangthyi* | KM024769 |
|  | *C. nigridia* | KM024772 |
|  | *C. niyomwanae* | KM024774 |
|  | *C. nuicamensis* | KM024775 |
|  | *C. omari* | KM024780 |
|  | *C. paripari* | KM024783 |
|  | *C. pemanggilensis* | KM024786 |
|  | *C. peninsularis* | KM024789 |
|  | *C. perhentianensis* | KM024821 |
|  | *C. phangngaensis* | KY091235 |
|  | *C. pseudomcguirei* | KM024825 |
|  | *C. psychedelica* | KM024827 |
|  | *C. punctatonuchalis* | KY091236 |
|  | *C. roticanai* | KM024830 |
|  | *C. selamatkanmerapoh* | KM024833 |
|  | *C. shahruli* | KM024834 |
|  | *C. siamensis* | KM024839 |
|  | *C. stongensis* | KM024840 |
|  | *C. sundainsula* | KM024847 |
|  | *C. temiah* | KM024849 |
|  | *C. thachanaensis* | KY091239 |
|  | *C. tucdupensis* | KM024855 |
|  | *C. vandeventeri* | KY091238 |
| *Alsophylax* | *Als. pipiens* | JX041309 |
| *Altiphylax* | *Alt. levitoni* | KC151974 |
|  | *Alt. stoliczkai* | KC151972 |
| *Cyrtodactylus* | *Cy. battalensis* | KC151983 |
|  | *Cy. pateni* | KU232620 |
|  | *Cy. phillippinicus* | JX440550 |
|  | *Cy. russelli* | JX440555 |
|  | *Cy. sharkari* | KJ659853 |
| *Gehyra* | *G. multiporosa* | JX524070 |
|  | *G. occidentalis* | JX524108 |
|  | *G. rohan* | KX954301 |
|  | *G. spheniscus* | JX524116 |
|  | *G. xenopus* | JX524129 |
| *Hemidactylus* | *H. flaviviridis* | AB937992 |
|  | *H. frenatus* | KM975950 |
|  | *H. garnotii* | EU268364 |
|  | *H. greefii* | EU268369 |
|  | *H. tenkatei* | KM975946 |
| *Hemiphyllodactylus* | *H. hongkongensis* | MF893333 |
|  | *H. linnwayensis* | MF576990 |
|  | *H. montawaensis* | MF576999 |
|  | *H. titiwangsaensis* | KF219795 |
|  | *H. tonywhitteni* | MF576992 |
|  | *H. typus* | KF219797 |
| *Microgecko* | *M. helenae* | JX041386 |
| *Perochirus* | *P. ateles* | JN393946 |
| *Stenodactylus* | *S. doriae* | KC151985 |
|  | *S. leptocosymbotus* | HQ443536 |
|  | *S. slevini* | KC151986 |
|  | *S. sthenodactylus* | KC151987 |
|  | *S. yemenensis* | HQ443550 |

**Table S2.** Dataset and literature review for logistic regression model.

| Species | Active_time | Sympatric *Cyrtodactylus* species | Habitat type | Max. SVL (mm) | Dispersal potential  (km) | Night temp. (°C) | Mean diurnal temp. range (°C) | Temp. seasonality (°C) | Mean temp. of coldest quarter (°C) | Precipitation seasonality (mm) | Precipitation of driest quarter (mm) | References |
| --- | --- | --- | --- | --- | --- | --- | --- | --- | --- | --- | --- | --- |
| *C. boulengerii* | Diurnal | Cyrto_Sym | Rock | 69.0 | 8.481 | 21.91 | 6.10 | 8.97 | 25.00 | 75.00 | 28.67 | Grismer et al. 2014 |
| *C. psychedelica* | Diurnal | Cyrto_Sym | Rock | 75.3 | 3.468 | 23.44 | 6.30 | 8.34 | 25.15 | 62.50 | 73.00 | Grismer et al. 2014 |
| *C. monachorum* | Diurnal | No_Cyrto | Rock | 35.1 | 26.278 | 22.94 | 9.20 | 4.61 | 25.95 | 54.50 | 155.00 | Grismer et al. 2009 |
| *C. biocellata* | Diurnal | Cyrto_Sym | Rock | 40.1 | 30.731 | 23.20 | 9.15 | 5.74 | 25.68 | 47.75 | 173.50 | Grismer et al. 2008 |
| *C. roticanai* | Diurnal | No_Cyrto | Others | 47.0 | 13.327 | 22.68 | 9.15 | 4.54 | 25.55 | 54.00 | 159.00 | Grismer & Onn 2010 |
| *C. affinis* | Diurnal | No_Cyrto | Others | 50.8 | 16.764 | 22.52 | 8.85 | 3.79 | 25.45 | 42.50 | 329.50 | Grismer et al. 2008 |
| *C. mahsuriae* | Nocturnal | No_Cyrto | Others | 36.6 | 10.286 | 23.22 | 9.15 | 4.62 | 25.75 | 54.50 | 158.00 | Grismer et al. 2015 |
| *C. chanthaburiensis* | Nocturnal | No_Cyrto | Others | 42.2 | 235.937 | 21.23 | 9.05 | 10.88 | 24.25 | 70.18 | 61.91 | Grismer et al. 2014 |
| *C. aurantiacopes* | Diurnal | Cyrto_Sym | Rock | 58.4 | 2.002 | 23.51 | 6.70 | 8.47 | 25.80 | 73.00 | 42.00 | Self-observation |
| *C. caudanivea* | Diurnal | Cyrto_Sym | Rock | 47.2 | 2.762 | 23.36 | 6.70 | 8.15 | 25.10 | 70.00 | 55.00 | Self-observation |
| *C. nuicamensis* | Diurnal | Cyrto_Sym | Rock | 48.2 | 17.165 | 22.65 | 7.13 | 8.03 | 25.80 | 67.33 | 46.67 | Self-observation |
| *C. tucdupensis* | Diurnal | Cyrto_Sym | Rock | 51.0 | 4.478 | 22.89 | 7.00 | 8.34 | 25.80 | 69.00 | 41.50 | Self-observation |
| *C. siamensis* | Nocturnal | No_Cyrto | Others | 39.7 | 614.854 | 21.73 | 8.68 | 10.20 | 24.40 | 64.00 | 122.60 | Grismer et al. 2010 |
| *C. omari* | Nocturnal? | No_Cyrto | Others | 41.3 | 52.230 | 22.43 | 9.00 | 6.16 | 25.65 | 48.50 | 168.50 | Grismer et al. 2014 |
| *C. chanardi* | Diurnal | No_Cyrto | Others | 40.1 | 199.326 | 21.15 | 9.20 | 7.91 | 24.30 | 53.17 | 190.00 | Grismer et al. 2010 |
| *C. shahruli* | Nocturnal | No_Cyrto | Others | 36.5 | 139.852 | 22.99 | 9.18 | 3.81 | 26.00 | 38.80 | 348.40 | Grismer et al. 2010 |
| *C. vandeventeri* | Nocturnal | No_Cyrto | Others | 44.7 | 154.023 | 21.30 | 8.83 | 7.03 | 24.67 | 70.00 | 127.00 | Grismer et al. 2010 |
| *C. flavigaster* | Diurnal | No_Cyrto | Rock | 50.1 | 18.144 | 23.09 | 9.70 | 3.65 | 25.10 | 27.00 | 424.00 | Onn & Grismer 2008 |
| *C. argus* | Diurnal | No_Cyrto | Rock | 65.2 | 19.690 | 20.54 | 7.75 | 6.82 | 21.85 | 53.00 | 394.50 | Grismer et al. 2014 |
| *C. karsticola* | Diurnal | No_Cyrto | Rock | 48.1 | 5.555 | 21.43 | 8.90 | 6.18 | 24.80 | 40.50 | 335.00 | Grismer et al. 2008 |
| *C. niyomwanae* | Nocturnal | No_Cyrto | Rock | 56.8 | 14.447 | 23.02 | 9.10 | 6.55 | 25.65 | 48.50 | 162.00 | Grismer et al. 2010 |
| *C. neangthyi* | Nocturnal | No_Cyrto | Rock | 54.0 | 88.614 | 21.79 | 8.45 | 9.74 | 24.85 | 72.50 | 52.50 | Grismer et al. 2010 |
| *C. harimau* | Diurnal | Cyrto_Sym | Others | 40.7 | 10.266 | 22.37 | 10.45 | 3.76 | 26.50 | 45.00 | 274.50 | Onn et al. 2010 |
| *C. pseudomcguirei* | Diurnal | No_Cyrto | Others | 42.5 | 68.846 | 20.67 | 8.83 | 3.94 | 22.43 | 30.67 | 532.67 | Grismer et al. 2009 |
| *C. huaseesom* | Nocturnal | No_Cyrto | Rock | 43.5 | 32.637 | 21.29 | 10.25 | 13.84 | 23.50 | 94.00 | 24.00 | Grismer et al. 2010 |
| *C. mcguirei* | Diurnal | Cyrto_Sym | Rock | 65.0 | 70.069 | 18.93 | 8.47 | 3.86 | 21.27 | 30.67 | 518.00 | Grismer et al. 2008 |
| *C. grismeri* | Diurnal | Cyrto_Sym | Rock | 50.6 | 5.526 | 20.21 | 9.50 | 4.43 | 24.15 | 33.50 | 442.00 | Wood et al. 2013 |
| *C. flavolineata* | Diurnal | No_Cyrto | Rock | 39.2 | 97.273 | 16.89 | 9.00 | 5.02 | 18.93 | 30.00 | 417.67 | Grismer et al. 2008 |
| *C. temiah* | Diurnal | No_Cyrto | Others | 46.7 | 7.009 | 22.34 | 7.00 | 7.16 | 25.25 | 40.50 | 346.50 | Grismer et al. 2014 |
| *C. narathiwatensis* | Diurnal | No_Cyrto | Rock | 53.2 | 50.842 | 20.49 | 8.80 | 6.05 | 24.70 | 43.50 | 304.50 | Grismer et al. 2010 |
| *C. hangus* | Diurnal | No_Cyrto | Rock | 50.5 | 1.974 | 22.12 | 10.05 | 6.06 | 24.35 | 26.00 | 447.50 | Grismer et al. 2014 |
| *C. selamatkanmerapoh* | Diurnal | Cyrto_Sym | Rock | 43.4 | 2.657 | 21.81 | 9.80 | 5.32 | 24.50 | 33.00 | 384.00 | Grismer et al. 2013 |
| *C. bayuensis* | Diurnal | No_Cyrto | Rock | 46.1 | 4.931 | 21.61 | 9.05 | 6.27 | 25.05 | 37.50 | 360.00 | Grismer et al. 2008 |
| *C. perhentianensis* | Diurnal | No_Cyrto | Rock | 47.0 | 2.589 | 22.44 | 7.30 | 8.34 | 25.30 | 75.00 | 304.00 | Grismer et al. 2014 |
| *C. limi* | Diurnal | No_Cyrto | Rock | 88.2 | 8.672 | 21.30 | 7.90 | 5.28 | 24.10 | 64.00 | 345.00 | Das&Grismer 2003 |
| *C. punctatonuchalis* | Nocturnal | No_Cyrto | Rock | 49.6 | 3.742 | 19.75 | 8.80 | 10.54 | 22.35 | 54.00 | 118.00 | Grismer et al. 2010 |
| *C. paripari* | Diurnal | Cyrto_Sym | Rock | 50.7 | 4.229 | 23.55 | 8.50 | 4.46 | 25.85 | 39.50 | 597.00 | Grismer&Onn 2009 |
| *C. sundainsula* | Diurnal | Cyrto_Sym | Rock | 84.5 | 4.371 | 22.10 | 6.50 | 5.60 | 25.10 | 27.50 | 445.00 | Grismer et al. 2014 |
| *C. kendallii* | Diurnal | Cyrto_Sym | Others | 58.4 | 888.222 | 22.91 | 8.22 | 3.72 | 25.46 | 33.15 | 607.62 | Grismer et al. 2014 |
| *C. pemanggilensis* | Diurnal | No_Cyrto | Rock | 76.0 | 3.842 | 23.28 | 7.73 | 5.56 | 25.33 | 61.33 | 380.33 | Grismer & Das 2006 |
| *C. baueri* | Diurnal | Cyrto_Sym | Rock | 67.4 | 4.481 | 22.00 | 7.65 | 5.52 | 25.35 | 55.50 | 409.50 | Das&Grismer 2003 |
| *C. mumpuniae* | Diurnal | Cyrto_Sym | Others | 56.6 | 38.074 | 22.28 | 6.55 | 5.73 | 25.30 | 28.50 | 450.50 | Grismer et al. 2014 |
| *C. peninsularis* | Diurnal | No_Cyrto | Others | 60.0 | 331.615 | 22.47 | 8.60 | 5.11 | 24.75 | 44.25 | 417.38 | Grismer et al. 2014 |
| *C. bidongensis* | Diurnal | No_Cyrto | Others | 58.1 | 0.857 | 23.12 | 7.10 | 8.59 | 25.50 | 77.00 | 308.00 | Grismer et al. 2014b |
| *C. kumpoli* | Nocturnal | No_Cyrto | Rock | 63.0 | 110.241 | 22.58 | 8.93 | 7.02 | 25.80 | 57.00 | 169.67 | Grismer et al. 2014 |
| *C. leucura* | Nocturnal | Cyrto_Sym | Rock | 62.7 | 5.373 | 20.65 | 8.65 | 2.86 | 23.70 | 27.50 | 540.00 | Kurita et al. 2017 |
| *C. lineogularis* | Diurnal | Cyrto_Sym | Rock | 38.0 | 14.880 | 24.24 | 8.35 | 13.12 | 25.40 | 66.50 | 83.50 | Wood et al. 2017 |
| *C. phangngaensis* | Diurnal | Cyrto_Sym | Rock | 42.0 | 3.833 | 22.78 | 8.40 | 6.69 | 26.10 | 60.00 | 164.50 | Wood et al. 2017 |
| *C. thachanaensis* | Diurnal | Cyrto_Sym | Rock | 39.0 | 3.794 | 23.44 | 9.50 | 10.09 | 25.40 | 52.00 | 154.00 | Wood et al. 2017 |
| *C. stongensis* | Nocturnal | No_Cyrto | Rock | 49.3 | 4.040 | 21.26 | 9.00 | 6.10 | 25.15 | 38.50 | 347.00 | Grismer et al. 2014 |
| *C. nigridia* | Diurnal | No_Cyrto | Rock | 75.5 | 66.749 | 22.35 | 8.33 | 4.17 | 25.30 | 52.33 | 493.00 | Grismer et al. 2014 |

**
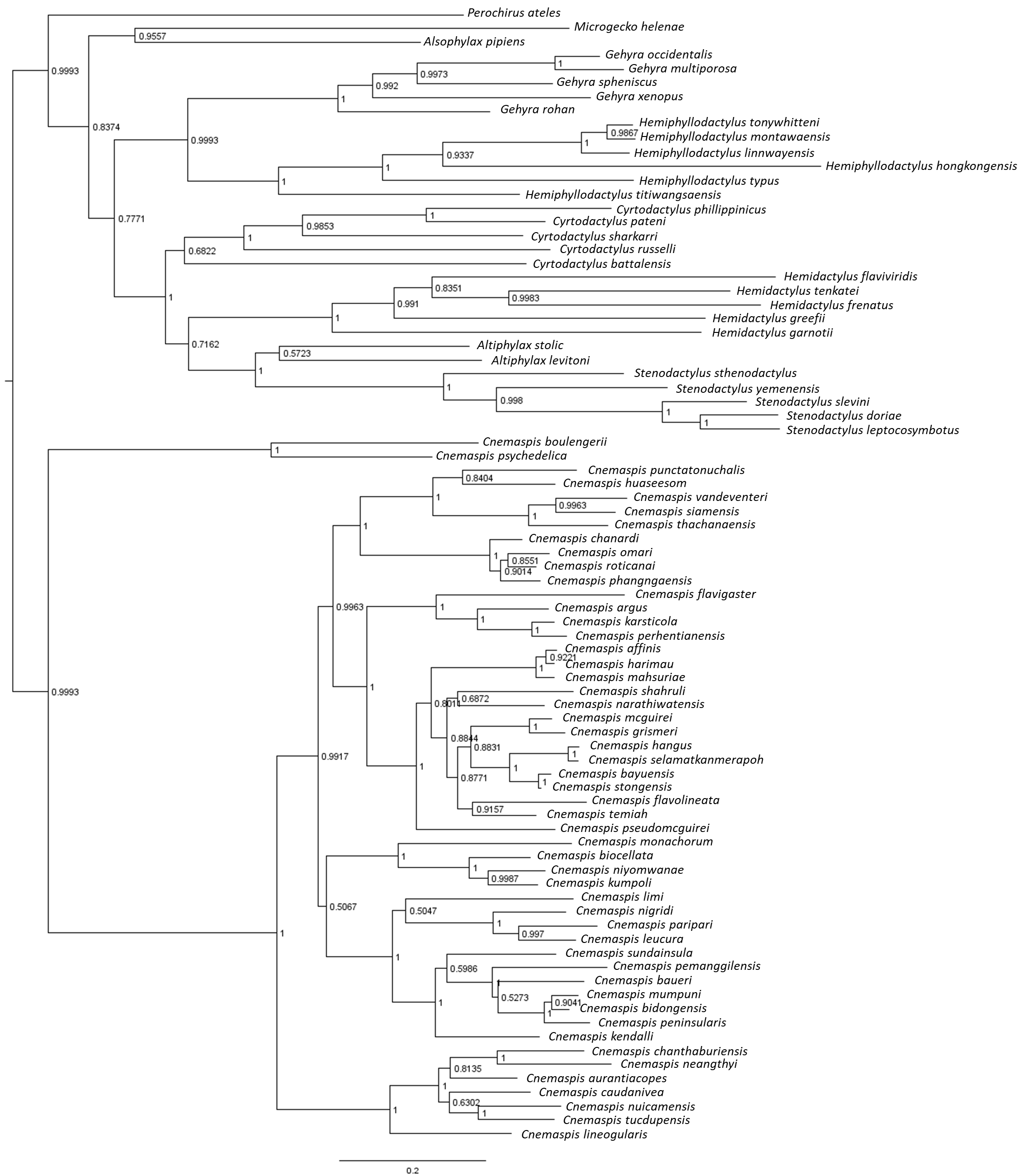
**

**Figure S1.** Phylogeny of *Cnemaspis* genus and outgroup from Bayesian analysis of mitochondrial gene dataset.

**Table S3.** Comparison among transition rates models used in the maximum-likelihood ancestral state construction for stochastic mapping method.

| Trait | Model | d.f. | ln likelihood | AIC scores |
| --- | --- | --- | --- | --- |
| Temporal activity | ARD* | 2 | -28.25734 | 60.51469 |
|  | ER | 1 | -32.86215 | 67.72429 |
| Habitat use | ARD* | 2 | -25.50891 | 55.01783 |
|  | ER | 1 | -27.89241 | 57.78482 |

*: AIC scores of the best fitting model
